# The Effects of Combinatorial Genistein and Sulforaphane in Breast Tumor Inhibition: Role in Epigenetic Regulation

**DOI:** 10.3390/ijms19061754

**Published:** 2018-06-13

**Authors:** Bidisha Paul, Yuanyuan Li, Trygve O. Tollefsbol

**Affiliations:** 1 Department of Biology, University of Alabama at Birmingham, Birmingham, AL 35294, USA; bidishap@uab.edu; 2 Comprehensive Cancer Center, University of Alabama at Birmingham, Birmingham, AL 35294, USA; lyy@uab.edu; 3 Nutrition Obesity Research Center, University of Alabama at Birmingham, Birmingham, AL 35294, USA; 4 Department of Pharmacology and Toxicology, University of Alabama at Birmingham, Birmingham, AL 35294, USA; 5 Comprehensive Diabetes Center, University of Alabama at Birmingham, Birmingham, AL 35294, USA; 6 Comprehensive Center for Healthy Aging, University of Alabama at Birmingham, Birmingham, AL 35294, USA

**Keywords:** genistein, sulforaphane, breast cancer, epigenetics, HDAC, KLF4, combination, synergy, broccoli, transgenic mouse model

## Abstract

Dietary compounds that possess the properties of altering epigenetic processes are gaining popularity as targets for cancer prevention studies. These compounds when administered at optimal concentrations and especially in combination can have enhanced effects in cancer prevention or therapy. It is important to study the interaction of two or more compounds in order to assess their role in enhancing prevention. Genistein (GEN), found in soy, has been extensively studied for its role as an epigenetic modifier especially as a DNA methyltransferase (DNMT) inhibitor and sulforaphane (SFN), found in cruciferous vegetables, is known as a histone deacetylase (HDAC) inhibitor. However, very little is known about the effects of these two compounds in conjunction in breast cancer prevention or therapy. In our current study, we determined that, at certain doses, the compounds have synergistic effects in decreasing cellular viability of breast cancer cell lines. Our results indicate that the combination of GEN and SFN is much more effective than their single doses in increasing the rate of apoptosis and lowering the colony forming potential of these cells. We determined that these compounds inhibit cell cycle progression to G2 phase in MDA-MB-231 and G1 phase in MCF-7 breast cancer cell lines. Additionally, we determined that the combination is effective as an HDAC and histone methyltransferase (HMT) inhibitor. Furthermore, we demonstrated that this combination downregulates the levels of HDAC2 and HDAC3 both at the mRNA and protein levels. We also found that these compounds have the potential to downregulate KLF4 levels, which plays an important role in stem cell formation. The combination of GEN and SFN is also effective in downregulating hTERT levels, which is known to be activated when KLF4 binds to its promoter region. Our hypothesis is further strengthened by *in vivo* studies, where the combination is administered to transgenic mice in the form of genistein and SFN-enriched broccoli sprouts. We have demonstrated that the combination is more effective in preventing or treating mammary cancer via extending tumor latency and reducing tumor volumes/sizes than either of these dietary components administered alone. These results are consistent with our *in vitro* study suggesting potential preventive and therapeutic effects of this novel dietary combinatorial approach against breast cancer.

## 1. Introduction

According to recent statistics, though the breast cancer death rate has decreased over the years, overall breast cancer incidence rates have increased among most races [1]. The cause for breast cancer is still unknown though most of the evidence points toward genetic and epigenetic abnormalities. Epigenetic gene alterations are an important hallmark of breast cancer. A common cause of breast cancer is epigenetic modifications, especially increasing DNA methylation and histone deacetylation. Bioactive dietary compounds are of special interest because of their anticancer properties and numerous studies have suggested that these dietary compounds can alter abnormal epigenetic states in cancer [2,3]. Regular consumption of bioactive nutritional compounds can alter the epigenome and play an active role in cancer prevention and therapy [4]. Several dietary compounds administered singly or in conjunction with other dietary compounds or other chemotherapeutic agents can reverse abnormal gene activation or silencing in cancer cells [5].

Combinatorial epigenetic marks are gaining importance as biomarkers for breast cancer diagnoses [6]. Choosing an optimum concentration of dietary combination that can decrease DNA methylation and deacetylation levels may be effective to synergistically activate tumor suppressors [7]. Thus, combinatorial regimens are gaining increasing popularity in cancer research because of their effectiveness in preventing and treating cancer. The combination of two or more compounds has been found to be more effective than compounds acting singly in many cases [8,9] by the use of a quantitative measure named Combination Index. Combination Index measures the degree of interaction among drugs in the form of synergism or antagonism for a given end point of the effect measurement [10]. The effectiveness of single compounds is too often performed at very high doses that can confer potential side effects. Lower doses of the single compounds in combination may have a better chemopreventive potential and translational impact. 

**Figure 1:**
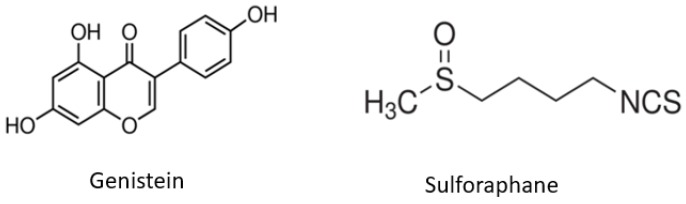
Structure of genistein (GEN) and sulforaphane (SFN). The chemical structures of GEN and SFN are displayed.

Genistein (GEN), found in natural soybean, is an isoflavone that is a known DNA methyltransferase (DNMT) inhibitor while sulforaphane (SFN), which is enriched in cruciferous vegetables such as broccoli sprouts (BSp) and kale, has been shown to have histone deacetylase (HDAC) inhibiting properties [11,12]. Figure 1 shows the structure of GEN and SFN. The inhibitory effects of GEN and SFN in tumor inhibition may be attributed to modulating epigenetic pathways by altering epigenetic marks in cancer cells. Our previous studies have shown that a combinatorial diet consisting of DNMT and HDAC inhibitors can alter the epigenetic machinery via direct and indirect inhibition of DNMTs and HDACs, thereby leading to modulating aberrations in DNA methylation and histone acetylation/deacetylation in tumor-related genes [13,14,15]. SFN is an inhibitor of HDAC activity, which results in an increase in global and local histone acetylation of a number of genes [16]. Unlike the single compounds, the combination of GEN and SFN has been understudied. GEN acts as an DNMT inhibitor reducing aberrant gene hypermethylation and SFN is an HDAC inhibitor that can remodel chromatin structure by affecting histone acetylation status in cancer cells and providing an open chromatin configuration.

We hypothesized that the optimal concentration of the two compounds should be effective in synergistically decreasing cancer cell viability. The correct combination of two or more dietary compounds that act synergistically can be very effective in preventing and treating breast cancer. Previous studies have shown that SFN exhibits chemopreventive activity by inducing cell cycle arrest and induction of cyclin-dependent kinase inhibitor 1A (p21 waf1/cip1) via KLF4 transcription factor mediation [17].

The role of KLF4 in breast cancer is ambiguous [18]. Downregulation of KLF4 in several cancers, such as colon, gastric, esophageal, prostate and lung may contribute to cellular hyperproliferation and malignant transformation, which is consistent with its role in cell cycle arrest and apoptosis [19,20,21]. KLF4 is thought to act both as a tumor suppressor and as an oncogene in breast cancer. However, KLF4 is highly expressed in more than 70% of breast cancers and functions as an oncogene [21]. It is also highly expressed in cancer stem cells [22]. Previous studies have shown that KLF4 is essential for promoting migration and invasion, resulting in tumor formation *in vivo* [20,23]. It is believed to promote tumor initiation through nuclear reprogramming [24]. Moreover, KLF4 has been shown to act in concert with HDAC2 and HDAC3. Another study has shown that KLF4 recruits epigenetic modifiers HDAC2 and HDAC3 at the *VEGF* promoter [25]. KLF4 also activates telomerase reverse transcriptase (TERT) expression and contributes to the maintenance of self-renewal in embryonic stem cells [26]. Since we found an optimum dosage using combinatorial GEN and SFN for decreasing cellular viability, we investigated the effect of this combination on KLF4 at the transcriptional as well as protein level. Recent studies have shown that the anticancer properties of benzyl isothiocyanate, a chemopreventive constituent derived from cruciferous vegetables, were enhanced by KLF4 knockdown in breast cancer cells [27]. We hypothesized that the combination of GEN and SFN causes breast cancer inhibition *in vitro* and *in vivo* via inhibition of KLF4 post-translationally as well as HDAC2 and HDAC3, which acts in conjunction with KLF4. Additionally, we sought to test whether the combination of GEN and SFN will also be effective in inhibiting hTERT, which is activated by recruitment of KLF4 to its promoter region. Moreover, we sought to evaluate whether a combinatorial GEN and SFN-enriched broccoli sprout diet could be effective in increasing tumor latency in spontaneous mouse models of mammary cancer and be more efficient in reducing tumor weight and volume as compared to mice groups administered a single compound or a control diet. 

## 2. Results

### 2.1. Effect of the Combination Treatment on the Cellular Viability of MCF-7 and MDA-MB-231 Breast Cancer Cells

**Figure 2:**
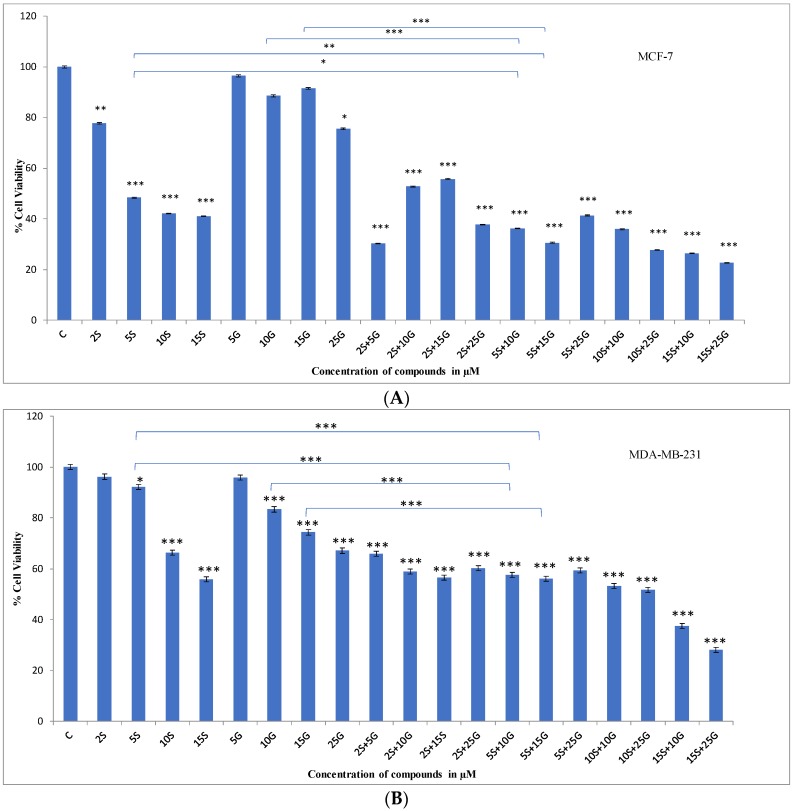

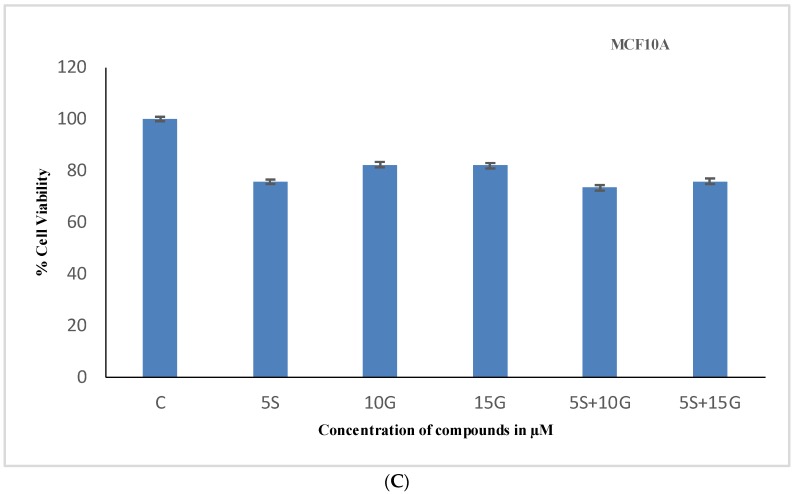
MTT assay. Combinatorial genistein (G) and sulforaphane (S) decrease cellular viability in breast cancer cells. (**A**) assays with 3-(4,5-dimethylthiazol-2-yl)-diphenyl tetrazolium bromide (MTT) of MCF-7 breast cancer cells were performed using several individual doses of GEN and SFN at different concentrations as well as their combination for 3 days; (**B**) MTT assays were performed on MDA-MB-231 cells for three days using the indicated concentrations; (**C**) MTT assays were performed on MCF10A cells for three days using the individual and combinatorial doses. The results represent three separately cultured experimental replicates. Numbers on the *x*-axis indicate compound concentrations (* *p* < 0.05, ** *p* < 0.01, *** *p* < 0.001, G, GEN; S, SFN). Data are presented as mean ± standard deviation (SD).

MTT (3-(4,5-Dimethylthiazol-2-yl)-2,5-Diphenyltetrazolium Bromide) assay was performed to determine the dose-dependent effects of the combination of GEN and SFN on the viability of breast cancer cells. Figure 2A–C show the effects of various doses of the dietary combination on MCF-7 and MDA-MB-231 breast cancer cell lines. In order to determine the optimum combination to be used for the study, the effect of the combinations was compared to their respective single doses with respect to their efficacy in decreasing cellular viability. CompuSyn software Version 1.0 (ComboSyn, Inc., Paramus, NJ, USA) [28] was used to determine the combination index (CI). The doses of 5 µM SFN + 10 µM GEN and 5 µM SFN + 15 µM GEN were found to be synergistically decreasing cellular viability in both breast cancer cell lines. Additionally, from previous studies, we know that 5 µM SFN and 10 µM or 15 µM GEN is not toxic to cells. We tested the effect of the combination in decreasing the viability of MCF10A non-cancerous cell lines and found that the combination does not significantly decrease the cellular viability of MCF10A cell lines. The lower doses of SFN and GEN were not as effective in decreasing cellular viability in combination and were not showing synergy in both the cell lines. The combination of 5 µM SFN + 10 µM GEN and 5 µM SFN + 15 µM GEN was effective in decreasing cellular viability in both cell lines compared to the dimethyl sulfoxide (DMSO) control. The combinatorial regimen was also synergistically effective in decreasing cellular viability when compared to their single doses. The combination of 5 µM SFN and 15 µM GEN had a CI <1, which is indicative of synergy [29]. This combination was extremely synergistic in MCF-7 cell lines having a CI = 0.295. Therefore, for our further experiments to study the effects at the molecular level, we decided to move forward with 15 µM GEN instead of 10 µM GEN. Table 1 shows the CI indices for some of the combinations. 

Table 1 shows the average values of combination index (CI) at 5.0 µM SFN and 10.0 µM GEN or 5.0 µM SFN and 15.0 µM GEN in MCF-7 and MDA-MB-231 breast cancer cell lines. The values of CI <1 are indicative of synergy between the two compounds, CI value = 1 indicates additive effects of the two compounds and CI value >1 indicate antagonism between the compounds. In MCF-7 cells, at a concentration of 5.0 µM SFN and 10.0 µM GEN, the CI value is in the range of synergism (0.3 < CI < 0.7) while at 5.0 µM SFN and 15.0 µM GEN, the CI values indicate strong synergism (0.1 < CI < 0.3). In MDA-MB-231 cell lines, for both the concentration of 5.0 µM SFN and 10.0 µM GEN as well as 5.0 µM SFN and 15.0 µM GEN, the CI values indicate synergism (0.3 < CI < 0.7). The CI values were calculated using the software CompuSyn Version 1.0 [30]. 

### 2.2. Combination of GEN and SFN Promote Cell Death as Evidenced through Cell Density Analysis and Apoptosis Assay

**Figure 3:**
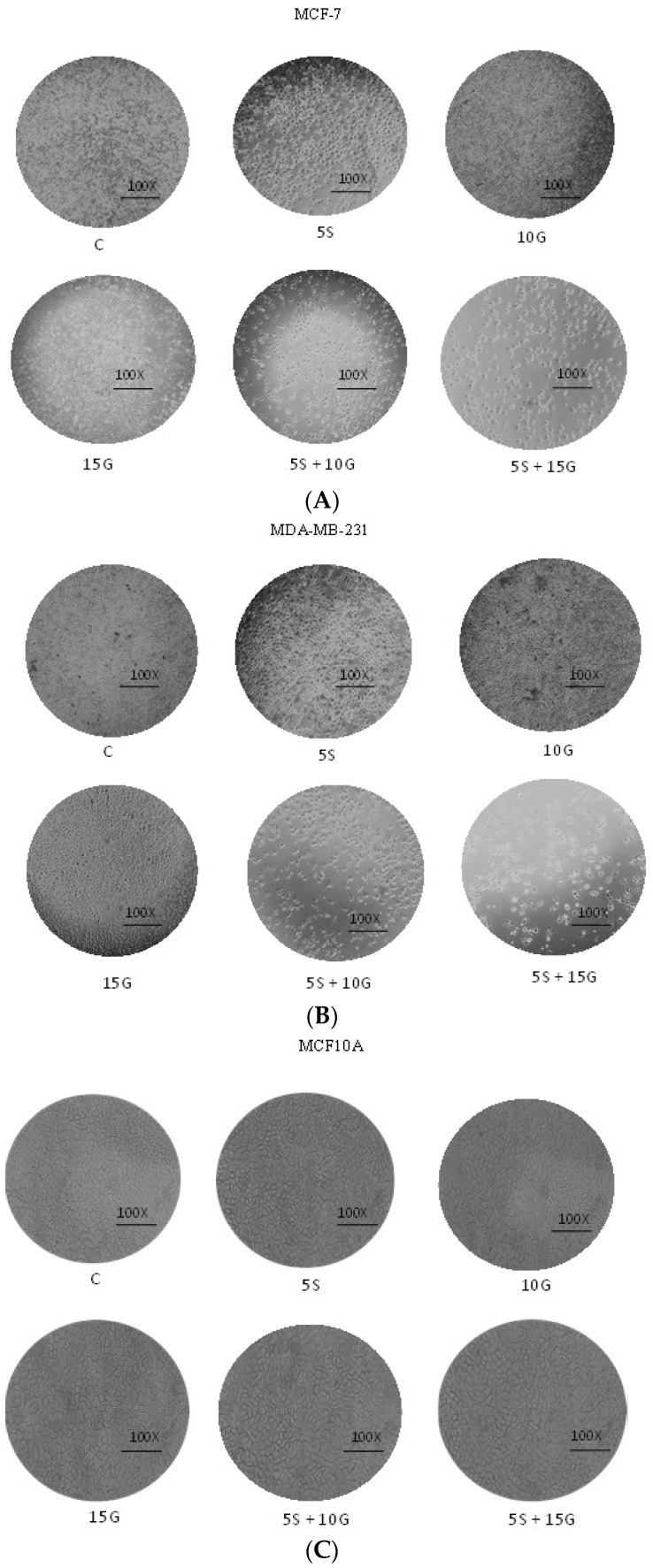
Cell density. Combinatorial GEN and SFN promote a decrease in cell density of breast cancer cells. (**A**) MCF-7 breast cancer cells were treated with 5.0 µM SFN and 10.0 µM GEN or 15.0 µM GEN singly and in combination for three days. Photographs were taken on the fifth day of culture; (**B**) MDA-MB-231 breast cancer cells were treated with 5.0 µM SFN and 10.0 µM GEN or 15.0 µM GEN singly and in combination for three days. Photographs were taken on the fifth day of culture; (**C**) MCF10A non-cancerous control cells were treated with 5.0 µM SFN and 10.0 µM GEN or 15.0 µM GEN singly and in combination for three days. Photographs were taken on the fifth day of culture. Dimethyl sulfoxide (DMSO) treatment served as control. Photos are representative of three replicates.

**Figure 4:**
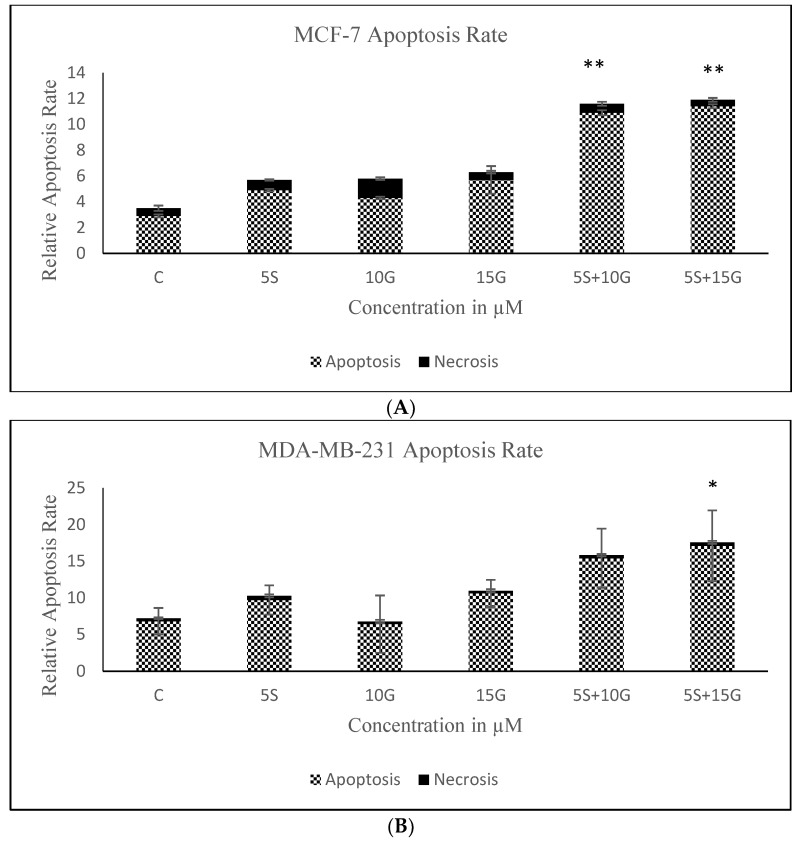
Apoptosis assay. Treatment with a combination of GEN and SFN promotes apoptosis in breast cancer cells (* *p* < 0.05, ** *p* < 0.01). (**A**) annexin V apoptosis analysis assay employing Fluorescence-activated Cell Sorting (FACS) analysis was performed on MCF-7 cells for three days at the indicated concentrations. The results represent three separate experimental replicates. Numbers on the *x*-axis indicate compound concentrations; (**B**) annexin V apoptosis analysis assay using FACS analysis was performed on MDA-MB-231 cells for three days at the indicated concentrations. The results represent three separate experimental replicates. Numbers on the *x*-axis indicate compound concentrations. Data are presented as mean ± SD.

As evidenced through cell density analysis (by capturing photos of cultured cells) in Figure 3, both MCF-7 and MDA-MB-231 cells show a decrease in cell density and an increase in surface area between cells with the incorporation of the predetermined optimal concentrations of 5 µM SFN and 15 µM GEN in comparison with the MCF10A non-cancerous control cells. Both cell lines show these compounds to be effective in promoting apoptosis after three days of treatment. Furthermore, Figure 4 demonstrates an increase in apoptosis with the incorporation of combinatorial treatment when compared to treatment with single compounds further strengthening the hypothesis that the combination of GEN and SFN is more effective in increasing the rate of apoptosis in breast cancer cells than the same doses of the singly-administered compounds. 

### 2.3. Effect of the Combination on Treatment Cell Cycle Arrest

**Figure 5:**
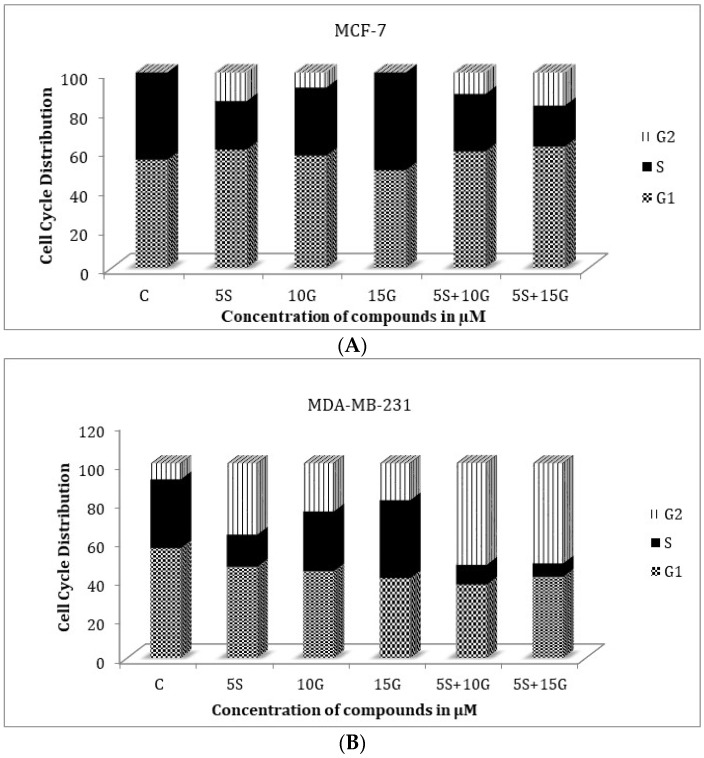
Cell cycle analysis. Combinatorial GEN and SFN arrests cells most abundantly at the G2 phase of the cell cycle for MDA-MB-231 cells and at the G1 phase for MCF-7 cells. (**A**) GEN and SFN arrest MCF-7 cells at the G1 phase of the cell cycle and prevents transition into M phase; (**B**) in MDA-MB-231 breast cancer cells, the combinatorial regimen arrests the cells at the G2 phase of the cell cycle.

Figure 5 demonstrates the effect of GEN and SFN on the cell cycle in ER (+) breast cancer cell line MCF-7 and ER (−) breast cancer cell line MDA-MB-231. Cells were treated with single doses of 5 µM SFN, 10 µM GEN or 15 µM GEN as well as the combination of 5 µM SFN and 10 µM GEN or 5 µM SFN and 15 µM GEN for three days. The combination of GEN and SFN revealed arrest in G1 phase of the cell cycle for the MCF-7 cells and an arrest at G2/M phase for the MDA-MB-231 cells. 

### 2.4. Combinatorial GEN and SFN Administration Decreases HDAC and HMT Expression

**Figure 6:**
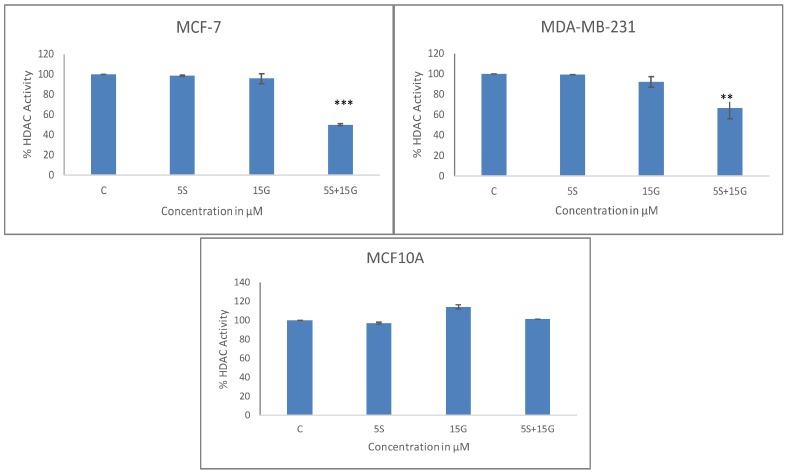
Histone deacetylase (HDAC) activity assay. HDAC enzymatic activity in breast cancer cells is downregulated by the combinatorial regimen. GEN and SFN downregulate HDAC activity in MCF-7 cells with the combination being the most effective. Combinatorial GEN and SFN downregulates HDAC activity in MDA-MB-231 cells with the combination being the most effective (** *p* < 0.01, *** *p* < 0.001). Combinatorial GEN and SFN does not significantly downregulate HDAC activity in MCF10A non-cancerous cells. Data are presented as mean ± SD.

**Figure 7:**
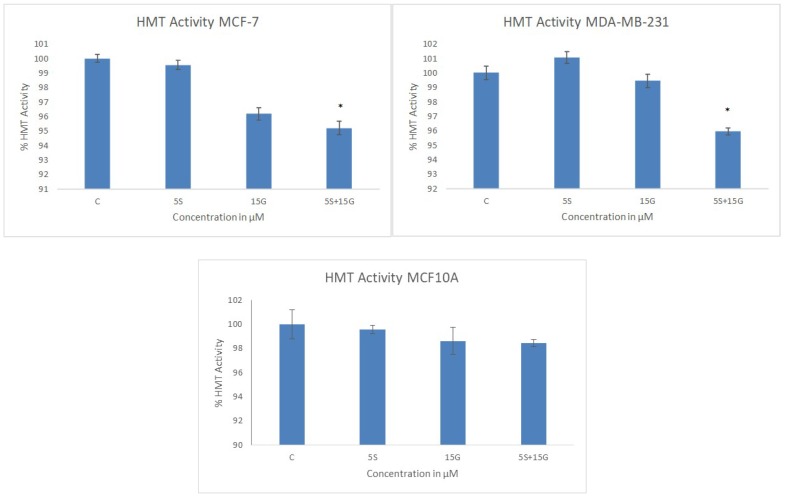
HMT activity. HMT enzymatic activity in breast cancer cells is downregulated by the combinatorial regimen: Combinatorial GEN and SFN downregulates HMT activity in MCF-7 cell lines with the combination being the most effective. GEN and SFN downregulate HMT activity in MDA-MB-231 cells with the combination being the most effective (*n* = 3; * *p* < 0.05). Combinatorial GEN and SFN does not significantly downregulate HMT activity in MCF10A non-cancerous cells. Data are presented as mean ± SD.

In order to observe the effect on epigenetic modifiers such as histone methyltransferases (HMTs) and HDACs, we treated the cells with the combination of GEN and SFN. In Figure 6, we found a significant decrease in enzymatic activity of HDACs in both cell lines. The combination treatment is more effective in the inhibition of overall HDAC activity. However, there was no significant downregulation of HDAC activity in the non-cancerous MCF10A cell line with the use of this combination. In Figure 7, we observed the effect of the combination of GEN and SFN in decreasing HMT activity in MCF-7 and MDA-MB-231 cell lines. The combination was effective in decreasing HMT activity more effectively than the DMSO control and single doses. There was, however, no significant decrease in HMT activity in the MCF10A control cell lines. 

### 2.5. GEN and SFN in Combination Induces Changes in KLF4, HDAC2 and HDAC3 mRNA Levels

**Figure 8:**
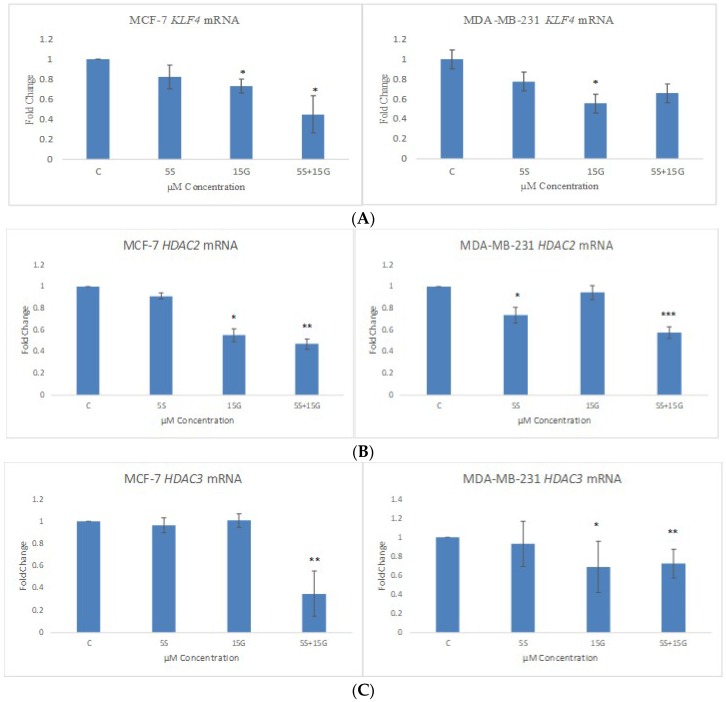
RT-PCR. *KLF4*, *HDAC2* and *HDAC3* mRNA levels are downregulated by the combinatorial treatment in both cell lines. Combinatorial GEN and SFN downregulate *KLF4*, *HDAC2* and *HDAC3* mRNA expression. qRT-PCR was performed to determine the mRNA expression. (**A**) *KLF4* mRNA expression is shown in MCF-7 and MDA-MB-231cells; (**B**) *HDAC2* mRNA expression is shown in MCF-7 and MDA-MB-231 cells; (**C**) *HDAC3* mRNA expression is shown in MCF-7 and MDA-MB-231 cells. The results represent the average of three separately cultured experimental replicates. (* *p* < 0.05, ** *p* < 0.01, *** *p* < 0.001).

KLF4 has an ambiguous role in breast tumor inhibition. Since KLF4 acts as an oncogene in most breast cancer cells, we decided to check the effect of the combinatorial regimen in downregulating the oncogene at the transcriptional level. As hypothesized, in MCF-7 cells, the combination of 5 µM SFN and 15 µM GEN was effective in downregulating KLF4 more effectively than their singly-administered doses at the mRNA level (Figure 8A). However, in the MDA-MB-231 cells, we did not observe a significant downregulation with the combination at the mRNA level. Since KLF4 works in concert with *HDAC2* and *HDAC3*, we sought to observe its effect on the two epigenetic modifying enzymes at the transcriptional level. As expected, the combination was effective in downregulating *HDAC2* and *HDAC3* at the transcriptional level. Figure 8B,C show the effect of the combination on *HDAC2* and *HDAC3* mRNA level.

### 2.6. Combination Treatment Induces Changes in KLF4, hTERT, HDAC2 AND HDAC3 Protein Levels

**Figure 9:**
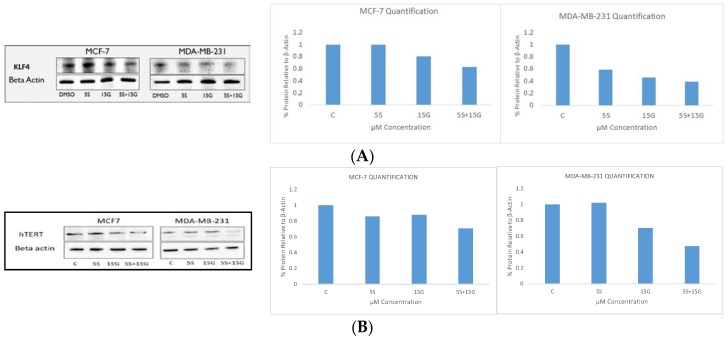
KLF4 Western blot. KLF4 protein levels are downregulated by GEN and SFN in breast cancer cell lines: (**A**) combinatorial GEN and SFN downregulates KLF4 protein expression in MCF-7 and MDA-MB-231 breast cancer cells with the combination appearing to be the most effective. Results of densitometry analyses of the aforementioned western blots using ImageJ software; (**B**) combinatorial GEN and SFN downregulates human telomerase reverse transcriptase (hTERT) protein expression in MCF-7 and MDA-MB-231 breast cancer cells. Results of densitometry analyses of the aforementioned Western blot using ImageJ software (version 1.51k, NIH, Bethesda, MD, USA). The figures are representative of similarly performed experiments (*n* = 3). The densitometry analysis was done of the representative figure.

**Figure 10:**
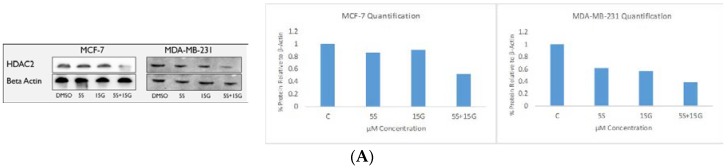

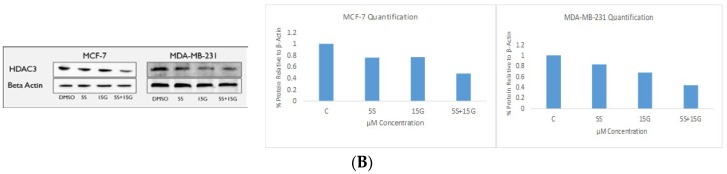
Western Blot. HDAC2 and HDAC3 protein levels are downregulated by GEN and SFN in breast cancer cells. (**A**) combinatorial GEN and SFN downregulates HDAC2 in MCF-7 and MDA-MB-231 breast cancer cells with the combination appearing to be the most effective. Results of densitometry analyses of the aforementioned western blots using ImageJ software; (**B**) combinatorial GEN and SFN downregulates HDAC3 expression in MCF-7 and MDA-MB-231 breast cancer cells with the combination appearing to be the most effective. Results of densitometry analyses of the aforementioned Western blots using ImageJ software. The figures are representative of similarly performed experiments (*n* = 3). The densitometry analysis was done of the representative figure.

Since KLF4 is known to be recruited to the promoter region of *hTERT* for its activation [31], we sought to evaluate the effect of the combination treatment on hTERT protein levels. We found that, the combination was effective in downregulating KLF4 protein levels and hTERT protein levels. Figure 9A shows that the combination is more effective than the single doses in inhibiting KLF4. The densitometry analysis of the image using imageJ software shows the quantitative analyses of the downregulation. Figure 9B shows the protein level downregulation of hTERT using the combination and the corresponding quantitative densitometry analyses of the images. Figure 10A shows the protein level downregulation of HDAC2 and HDAC3 which is believed to act in concert with KLF4. Since KLF4 is downregulated by the combinatorial regimen, we tested for downregulation of HDAC2 and HDAC3. The western blot analyses show downregulation of HDAC2 (Figure 10A) and downregulation of HDAC3 (Figure 10B) at the protein level, the downregulation being most effective at 5 µM SFN and 15 µM GEN doses.

### 2.7. Combinatorial Dietary Regimen of GEN and Broccoli Sprouts (BSp) Is Effective in Reducing Mammary Tumor Incidence and Delaying Tumor Latency in a Spontaneous Breast Cancer Mouse Model

In order to test the efficacy of the combinatorial dietary regimen of GEN and BSp in an *in vivo* model system, we treated the transgenic mouse model, C(3)1-SV40, that can spontaneously develop mammary cancer in early age, with either GEN and BSp alone or with a combination treatment of these diets. The special diets were supplemented with 250 mg/kg of GEN and/or 13% BSp in corn-oil-based AIN-93G diet from four weeks of age and continued throughout the study, which mimics the processes of prevention and therapeutic effects by these treatments. The concentrations of the diets used in this study were corresponding to ~2 g of isoflavone intake and/or consuming 133 g (~2 cups) broccoli sprout/per day by an adult human, respectively.

**Figure 11:**
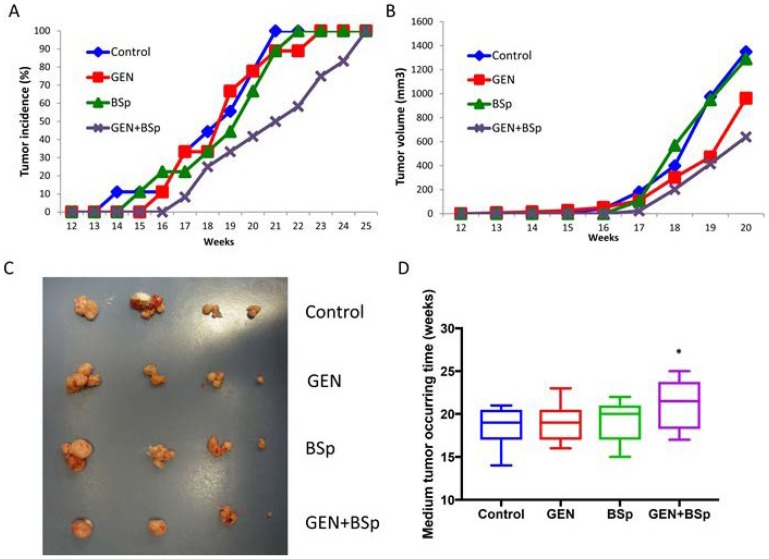
Tumor data analysis. Tumor inhibitory effects of dietary GEN and broccoli sprouts (BSp) on tumor growth of the ERα (−) transgenic mammary cancer C3(1)-SV40 (SV40) mouse model. Female SV40 mice (9–12 per group) were administered either regular control diet, GEN diet (250 mg/kg), 13% BSp diet or the two diets in combination from four weeks of age until termination of the experiment. Tumor volumes and sizes were observed weekly. (**A**) tumor incidence; (**B**) tumor volumes; (**C**) representative photographs of mammary tumors in different experimental groups when harvested at the termination of the experiment; (**D**) tumor latency. * *p* < 0.05, significantly different from the control group.

We found that the combination treatment of GEN and BSp was the most effective in reducing tumor incidence and tumor volume as compared to the control and either single treatment alone during the whole course of the experiment (Figure 11A,B). Figure 11C shows the actual tumor comparison in different treatment groups at the endpoint and the combination treatment resulted in the smallest tumor sizes compared to the other treatment groups. More importantly, our results indicate that only the combinatorial treatment with GEN and BSp can lead to a significant delay of tumor latency (*p* < 0.05). These results suggest that the combination treatment with soybean GEN and SFN-enriched BSp are more effective in both preventing mammary cancer via extending tumor latency and reducing tumor volumes/sizes than either of these treatments alone. These results are consistent with the *in vitro* study suggesting potential preventive and therapeutic effects of this novel dietary approach on breast cancer.

## 3. Discussion

The side effects of toxic drugs and chemotherapy have led to a growing interest in compounds that provide non-toxic and safer therapy. The use of dietary compounds as chemotherapeutic and chemopreventive agents is becoming very popular as an additional form of therapy. A healthy diet still remains among the most effective chemoprevention methods [32]. At the molecular level, a healthy diet consisting of bioactive nutritional compounds ensures proper epigenetic regulation of the system, which can facilitate prevention of cancer. Breast cancer is one of the leading causes of cancer death in women. The most prominent epigenetic features altered in breast cancer are DNA methylation patterns and chromatin states [33]. Therefore, the study of bioactive dietary compounds has gained prominence because they may have the ability to modulate epigenetic aberrations in cancer and restore the normal epigenetic state of oncogenes or tumor suppressor genes [34]. 

Breast cancer statistics have revealed that the incidence of breast cancer is much lower among Asians as compared to Caucasians and African Americans [35]. Previous studies have attributed this variation in breast cancer incidence to differences in diet and lifestyle [36]. The Asian diet in general is rich in soy and cruciferous vegetables and as a result may provide protection against breast cancer [37]. Our current study focuses on assessing the impact of consumption of GEN and cruciferous vegetables in breast tumor prevention.

GEN, which is an isoflavone found in soybeans, is an anticancer agent due to its property of being a DNMT inhibitor. GEN has also shown HDAC inhibiting properties. Additionally, GEN has been known to resensitize breast cancer cells to estrogen-targeted chemotherapy by activation of estrogen receptor in ER-negative breast cancer [38]. GEN has been known to block breast cancer cells in G2/M phase of the cell cycle by induction of p21 WAF1/CIP1 expression [39] and increase acetylation of histones 3 and 4 and H3-lysine4 at the *p21 WAF1/CIP1* and *p16 INK4a* transcription start sites, mediated by induction of histone acetyltransferases (HATs) in prostate cancer cells [40]. In renal cancer, GEN increases histone acetylation by enhancing HAT activity and causes acetylation of histones 3 and 4, dimethyl-H3K4 and trimethyl-H3K4 near the transcription start site at the *BTG3* gene promoter [41]. Our lab has recently shown that GEN alters the gut microbiome of breast cancer patients possibly via epigenetic modulation brought about by metabolites produced by bacteria residing in the gut [42]. SFN, which is abundant in cruciferous vegetables such as broccoli or kale, is an HDAC inhibitor and is known to be an effective inhibitor of several class I and II HDAC inhibitors [43]. It inhibits breast cancer stem cells *in vitro* and *in vivo* by suppressing the Wnt/ β-catenin pathway [44,45]. Our earlier studies have shown that SFN can cause epigenetic repression of hTERT expression in breast cancer cells [46]. It exhibits chemopreventive properties by inducing cell cycle arrest via induction of cyclin-dependent kinase inhibitor 1A (p21^WAF1/CIP1^). Higher levels of HDAC1 and HDAC2 expression are significantly associated with tumor differentiation [47]. Previous studies have shown that SFN binds to the HDAC pocket and inhibits HDAC activity [48]. In our current study, we used a combination of an HDAC inhibitor with the use of SFN along with a DNMT inhibitor with the use of GEN as this approach may have more tumor-inhibiting potential. Our rationale for using this combination is that the HDAC inhibitor will cause acetylation of histones and as a result a more open chromatin configuration and thus provide more accessibility to the DNA for the DNMT inhibitor to act upon. 

Here, we report for the first time that at optimal concentrations, the combination of GEN and SFN can have synergistic effects in reducing the viability of breast cancer cells with no significant effect on the viability of non-cancerous control cells. We have used a much lower than usual concentration of GEN and SFN where they effectively decrease the viability of breast cancer cells. The study is particularly important as we are able to show that in MCF-7 ER (+) and MDA-MB-231 ER (−) breast cancer cells, the combination is much more effective than singly administered doses of these compounds both *in vitro* and *in vivo* studies. At a concentration of 5 µM SFN and 15 µM GEN, the cellular viability of breast cancer cells decreases synergistically. The combination is effective in promoting apoptosis much more effectively than separate doses. Cell cycle analyses reveal that the cells are arrested at G2/M phase for MDA-MB-231 cells and at G1 phase for MCF-7 cells. The molecular mechanisms reveal that at the transcriptional level, the combinatorial regimen can decrease the levels of HDACs and a similar observation can be seen at the post-translational level. Upon further assessing HDACs and HMTs, it can be observed that the activity of HDACs and HMTs is decreased with the combination therapy. However, the combination was not effective in synergistically downregulating DNMT activity. In addition to being a DNMT inhibitor, GEN is also known to possess histone modifying properties [40,41,49]. Previous studies have shown that GEN increased acetylated histones 3, 4, and H3/K4 at the *p21* and *p16* transcription start sites and also increased the expression of histone acetyltransferases that function in transcriptional activation [50]. Thus, the combination of GEN and SFN acting synergistically may be due to the histone acetyltransferase promoting activity of GEN that activates tumor suppressor genes such as *p21* and *p16*, whereas SFN may be acting as an HDAC inhibitor and suppressor of the activity of oncogenes. Together the combination of GEN and SFN is effective in altering epigenetic modifications in breast cancer cell lines. In our current study we observed the downregulation of kruppel like factor-KLF4 using this combination. Additionally, we have observed that *in vivo* the combination was much more efficacious in reducing tumor volume and causing tumor latency in mice that that were fed the combinatorial diet as compared to the mice on a control diet or the diets consisting of the singly administered compounds.

In our study, we sought to assess the role of dietary phytochemicals in breast tumor inhibition. Therefore, our goal was to use compounds in their natural form. However, it was not possible to use soy to assess the efficacy of GEN since soy contains a mixture of compounds such as diadzein, glycetein and genistein. Therefore, it is difficult to elucidate the role of GEN in this complex mixture. Hence, we chose to use the purified compound. However, broccoli sprouts contain 50 times higher concentration of glucoraphanin, which is converted to isothiocyanate by the enzyme myrosinase [51,52]. Thus, the use of broccoli sprout would be effective in assessing the role of SFN. Therefore, we used broccoli sprout instead of the purified compound. The dose of broccoli sprouts used is 13% BSp which is equivalent to 0.45% of SFN or 350 ppm. Studies in colorectal cancer have shown that dietary intake of 300 ppm SFN prevent adenoma formation in mice [53]. Further studies have shown that *in vivo* 250 mg/kg GEN was effective in mammary cancer chemoprevention [54,55].

Kruppel-like factors are transcriptional regulators that influence proliferation and several cellular functions in cancer. One family member, KLF4, can function both as a tumor suppressor and as an oncogene. KLF4 can activate and/or repress transcription depending on which transcription factors it interacts with and also on its target gene. Studies have indicated that p21 acts like a switch and modulates the activity of KLF4 [56]. Some studies have reported that KLF4 activates telomerase reverse transcriptase (TERT) expression and contributes to the maintenance of self-renewal in embryonic stem cells [57,58]. Recent studies have indicated that poly (ADP-ribose) polymerase (PARP1) interacts with KLF4, where knockdown of PARP1 reduces hTERT expression [26]. PARP1 recruits KLF4 to activate telomerase expression in cancer cells. GEN has been previously shown to decrease the expression of PARP1 [59]. In our current study we observed a downregulation of *KLF4* at the mRNA level for the MCF-7 cell line with the combination treatment. The combination was effective in downregulating KLF4 levels post-translationally for both MCF-7 and MDA-MB-231 cell lines. Previously, it has been reported that KLF4 expression is required to prevent epithelial-to-mesenchymal transition in MDA-MB-231 mammary cell line. The forced expression of KLF4 suppressed cell migration and invasion by restoring E-cadherin expression [60]. Therefore, we assessed the level of E-cadherin in the treated cells and observed an upregulation of E-cadherin in the cells treated with the combinatorial regimen (Figure S1). This may suggest that the upregulation could be independent of KLF4 downregulation. Previous studies have shown that GEN and SFN alone have increased the expression of E-cadherin through mechanisms not related to KLF4 regulation. GEN increases E-cadherin levels *in vivo* by directly targeting it and regulating the Wnt signaling pathway and prevented accumulation of β-catenin in the cytoplasm [61]. SFN has also been known to increase E-cadherin expression and inhibit prostate carcinogenesis and lung metastases in TRAMP (transgenic adenocarcinoma of the mouse prostate) mouse models along with an increase in apoptotic bodies [62]. Thus, the reports point towards an alternative pathway by the dietary phytochemicals to increase E-cadherin expression in cancer cells which may not necessarily involve KLF4 mediation. However, an in-depth study is warranted to study the association between KLF4 and E-cadherin expression in breast cancer cells.

The combination was also effective in downregulating HDAC2 and HDAC3 that act in concert with KLF4. Since KLF4 activates hTERT, with the downregulation of KLF4, hTERT should also be downregulated. Therefore, we assessed the levels of hTERT in breast cancer cells. As hypothesized, hTERT levels were significantly reduced using the combinatorial regimen in both of the breast cancer cell lines we tested.

The combinatorial dietary regimen of GEN and SFN was also administered to an ER (−) transgenic mammary cancer mouse model by orally feeding the mice with either GEN or SFN-enriched broccoli sprouts (BSps) diets alone or in combination. Our results showed combinatorial treatment with GEN and BSp diet led to the most effective response in both preventive and therapeutic effects on inhibition of mammary tumor *in vivo* as compared to either non-combinatorial treatment or control diet. The dietary combinatorial regimen consists of relatively low concentrations of GEN and SFN that are physiologically accessible by daily consumption of soy and BSp [63,64]. Thus, our novel combination dietary regimen shows an important bioavailability and translational potential toward chemoprevention and/or treatment of breast cancer. It would be interesting to design studies in the future to assess the influence of the combinatorial dietary regimen and compare their effects in ER positive, ER negative and BRCA1 mutated triple negative breast cancers.

## 4. Materials and Methods

**Cell Lines**: We used the ERα-positive MCF-7 and ERα-negative MDA-MB-231 breast cancer cells for this study. MCF10A human mammary epithelial cells were used as a non-cancerous control. All cell lines were obtained from the ATCC (American Type Culture Collection, Manassas, VA, USA). MCF-7 cells were derived from a 69-year-old Caucasian female from an excision of a chest wall nodule and from a pleural effusion [65]. MDA-MB-231 cells are relatively aggressive in comparison to MCF-7 cells and have a mutated p53 status [66]. MCF10A are non-tumorigenic cells derived from benign proliferative breast tissue and are spontaneously immortalized. They do not express estrogen receptors [67].

**Chemicals**: Genistein (≥95% pure), C_15_H_10_O_5_ with a molecular weight of 270.241 g/mol was purchased from Sigma-Aldrich (St. Louis, MO, USA) and R,S-sulforaphane (≥98% pure), C_6_H_11_NOS_2_ with a molecular weight of 177.28 g/mol, was purchased from LKT Laboratories, Minneapolis, MN, USA. Each compound was diluted in DMSO and stored in stocks of 10 mmol/L at −20 °C. 

**Cell Culture and Treatment**: The media to culture the MCF-7 and MDA-MB-231 cells was made using Dulbecco’s Modified Eagle’s Medium DMEM 1X (Corning cellgro-Thermo Fisher Scientific, Waltham, MA, USA), 10% by volume of fetal bovine serum (Atlanta Biologicals, Lawrenceville, GA, USA) and 1% by volume of 50X penicillin-streptomycin (Corning cellgro). The media for culturing MCF10A cells was made using DMEM F12 media (Corning cellgro-Thermo Fisher Scientific, Waltham, MA, USA), 5% Donor Horse Serum (Corning cellgro-Thermo Fisher Scientific, Waltham, MA, USA), 100 unit/mL penicillin streptomycin, 100 µL of 0.05 µg/mL hydrocortisone, 100 µL of 20 ng/mL EGF, 0.292 g of 2 mmol/L l-glutamine and 100 ng/mL cholera endotoxin. Incubation was done in an incubator that maintained a temperature of 37 °C and provided an environment having at 5% CO_2_ and 95% air. Cells were sub-cultured when they were about 90% confluent, counted using a hemocytometer and seeded into 6-well plates. After 24 h, they were treated with individual as well as combination doses of GEN and SFN for 3 days. DMSO was used as a control. Media and compounds were replaced every 24 h during the 3-day treatment. 

**Cell Density**: For the cell density analyses, approximately 200,000 cells were plated in 6-well plates. They were incubated for 24 h and then treated with GEN and/or SFN each day at the same time for 72 h. Media was replaced each day. On day four, cells were viewed under a microscope and images were taken at 100× magnification.

**MTT Assay**: MTT assay was performed to assess the percentage of viability of breast cancer cells and the control cells after treatment with GEN and SFN. Tetrazolium, 3-(4,5-dimethylthiazol-2-yl)-diphenyl tetrazolium bromide (MTT) was obtained from Sigma Aldrich (St. Louis, MO, USA) and diluted to 1mg/mL using PBS (Phosphate buffered saline). Cells were seeded in 96 well plates and treated with appropriate concentrations of GEN and SFN for 3 days. On the fourth day, 50 µL of MTT solution was added to each well and incubated for approximately 4 h at 37 °C. MTT solution was removed and DMSO was added and incubated for about 15 min. The color of the solution changed to shades of purple. The absorbance was measured using a spectrophotometer (BioRad; Hercules, CA, USA) at 595 nm.

**RNA Isolation**: RNA was isolated using the RNeasy kit from Qiagen (Valencia, CA, USA). MDA-MB-231 and MCF-7 cells were plated in 6-well plates and allowed to adhere to the plate for 24 h. The cells were then treated with individual and combination doses of GEN and SFN for 3 days. On the fifth day, the cells were collected using trypsin. After the cells detached from the plate, fresh media was added and then centrifuged at 300× *g* for 5 min. The pellets were used for further analysis using manufacturer’s protocol. 

**Quantitative Real-Time PCR**: cDNA synthesis kit (Bio-Rad) was used to reverse transcribe RNA (made using the protocol above) to cDNA. For the PCR setup, 1 µL of cDNA was used, along with 5 µL of SYBR green, 2 µL of nuclease-free water, 1 µL of reverse and forward primers specific for *KLF4*, *HDAC2* and *HDAC3*. The total volume was made upto10 µL. PCR was performed using CFX Connect Real Time system (Bio-Rad) where the thermal cycling initiation (4 min at 94 °C) preceded 35 cycles of PCR (94 °C, 15 s; 60 °C, 30 s, 72 °C, 30 s). GAPDH (Glyceraldehyde 3-phospate dehydrogenase) was used as an endogenous control. Fold change was calculated using ΔΔCq method [68]. All of the primers were purchased from Integrated DNA Technologies (Coralville, IA, USA). The primer sequences used were as follows:

GAPDH F 5′-ACC ACA GTC CAT GCC ATC AC-3′, R 5′-TCC ACC CTG TTG CTG TA-3′; KLF4 F 5′-CGA ACT CAC ACA GGC GAG AA-3′ R 5′-CGG AGC GGG CGA ATTT-3′; HDAC2 F 5′-CCG GAT CCA TGG CGT ACA GTC AAG GA-3′ R 5′-CCG CGG CCG CTC AAG GGT TGC TGA GT-3′; HDAC3 F 5′ TTG AGT TCT GCT CGC GTT ACA-3′ R 5′ CCC AGT TAA TGG CAA TAT CAC AGAT 3′

**Cell Cycle Analysis**: Approximate 5 × 10^4^ cells were plated in 6-well plates. Furthermore, 2 mL of media was added to the wells and allowed to attach to the surface of the plate for 24 h. After 24 h, the cells were treated with GEN and SFN for 3 days at approximately the same time each day. On the fifth day, the cells were collected and fixed with 70% ethanol. Cells were prepared for analyses with a flow cytometer by treating them with propidium iodide (0.04 mg/mL), 0.1% TritonX-100 and RNase (100 mg/mL). Fluorescent signals were read at the UAB Comprehensive Flow Cytometry Core.

**Annexin V Apoptosis Assay by Fluorescence-activated Cell Sorting (FACS)**: MCF-7 and MDA-MB-231 cells were seeded in 6-well plates. After 24 h, the cells were attached to the bottom of the well. The cells were then treated with the GEN and SFN for 3 days. On the fifth day, the cells were collected and prepared for apoptosis assay by using the Annexin V-conjugated Alexafluor 488 Apoptosis assay kit. Cells were treated with Alexa 488 and propidium iodide. The treated cells were then sent to UAB Comprehensive Flow Cytometry Core where they were analyzed using FACS-Caliber instrument (BD Biosciences, San Jose, CA, USA). The cells in Q1 phase were used in analysis of necrosis, whereas the ones in Q2 (early stage apoptosis) and Q4 (late stage apoptosis) phase were used for apoptotic analysis. 

**Western Blot Analysis**: Breast cancer cells were treated with individual doses of GEN and SFN and their combination for 3 days, followed by extraction of protein by RIPA (Radioimmunoprecipitation buffer) lysis buffer. The concentration of protein was then determined by performing Bradford Assay using Bio-Rad protein assay kit (Bio-Rad; Hercules, CA, USA). 4–15% Tris-HCL gel was used for electrophoresis. The wells were loaded with equal concentration of protein mixed with loading buffer. After separation of the proteins by electrophoresis (200 V), the protein was transferred onto nitrocellulose membrane using the Trans Turbo Blot (BioRad). After successful transfer, the membrane was blocked with blocking buffer using a MilliPor SnapID. The milk buffer was made using dry milk powder (0.5%) and Tween (1%) dissolved in Tris Buffered Saline solution. Primary antibodies (Cell Signaling Technology, Danvers, MA, USA) specific to the genes of interest was incubated for 20–30 min and washed for four times using 30 mL of TBST (1X Tris-buffered saline solution with 1% Tween). Secondary antibodies were then added for 15–20 min followed by washing three-four times with TBST. Chemiluminescent substrate was added to the blot according to manufacturer’s suggestion and images were captured using an imager (BioRad Chemidoc XRX+ system). 

**HDAC Activity Assay**: Breast cancer cells MCF-7 and MDA-MB-231 as well as control cells MCF10A were treated for 3 days with individual doses (Control, 5 µM SFN, 15 µM GEN) as well as their combination doses (5 µM SFN + 15 µM GEN). After treatment, the cells were collected and the nuclear extract was obtained using an EpiQuik Nuclear Extraction Kit from EpiGenTek (Farmingdale, NY, USA). An EpiQuik HDAC Activity/Inhibition Assay Kit obtained from EpiGentek (Catalog # P-4002) was used to colorimetrically measure HDAC activity using the protocol provided by the manufacturer.

**HMT Activity Assay**: Breast cancer cells MCF-7 and MDA-MB-231 as well as control cells MCF10A were treated for 3 days with single doses (Control, 5 µM SFN, 15 µM GEN) as well as their combination doses—5 µM SFN + 15 µM GEN. After treatment, the cells were collected and the nuclear extract was obtained using EpiQuik Nuclear Extraction Kit from EpiGenTek (Farmingdale, NY, USA). EpiQuik Histone Methyltransferase Activity/Inhibition Assay Kit (H3-K27) obtained from EpiGentek (Catalog # P-3005) was used to colorimetrically measure HMT activity using the protocol provided by the manufacturer. 

**Statistics and Data Analysis**: Any conclusion in the study was derived from three independent experimental replicates. Data were represented as mean + SD. Statistics for the study were performed using Microsoft Excel. Significance was determined using a Student’s *t*-test in Microsoft Excel. *p* < 0.05 (indicated by *) is considered statistically significant. All comparisons were done with the DMSO control. For evaluating the nature of interaction between GEN and SFN, CompuSyn software was used. The dose of each compound was entered individually since they are non-constant ratio combos. The CompuSyn software gives combination index (CI) values. CI < 1 indicates synergism between the compounds, CI = 1 indicates additive interaction between the compounds and CI > 1 indicates antagonism between the compounds. The interaction between the dietary phytochemicals is represented in the form of a graph called isobologram. CI index analyses are similar to isobologram analysis and provide a quantitative assessment of the interaction between drugs. The isobologram equation is a special case of the combination index equation. The isobologram assesses the interaction between the compounds at median effect concentration.

**Ethics**: The *in vivo* experiments using mouse models were performed by strictly adhering to the rules and guidelines of UAB’s Institutional Animal Care and Use Committee (IACUC-131009970 21-Oct-2013).

**Animal Experimental Design**: The C3(1)-SV40 Tag transgenic mouse model (SV40) was used to study the efficacy of the combination treatment of GEN and SFN. The SV40 mouse model can spontaneously develop hormone-independent invasive mammary cancer (ERα-negative breast cancer) at an early age of around 20 weeks. The SV40 mice at 4 weeks of age were randomly divided into different experimental groups (9–12 mice/group) and diets were administered from 4 weeks of age and continued throughout the study.

The experimental groups were as follows: Group (1). Control group: Mice were fed with modified corn oil-based AIN-93G diet; Group (2). GEN group: Mice were fed with 250 mg/Kg GEN diet; Group (3). BSp group: mice were fed with 13% BSp diet (*w*/*w*); (4). GEN + BSp group: Mice were administered the combined GEN and BSp diet with concentrations as described above. All diets were prepared using a modified corn oil-based AIN-93G diet as a base by a commercially available company (TestDiet, St. Louis, MO, USA).

Tumor volumes and body weight were measured weekly. Tumor volumes were calculated using the formula: tumor volume (cm^3^) = (length × width^2^) × 0.523 [69]. The experiment was terminated when the mean of tumor diameter in the control mice exceeded 1.0 cm. At the end of the experiment, the primary mammary tumors were excised, weighed and appropriately stored in liquid nitrogen for further analysis. 

## 5. Conclusions

In conclusion, the current study highlights the efficacy of this combinatorial diet consisting of GEN and SFN in enhancing the translational chemopreventive potential during breast tumorigenesis. *In vitro*, the combination has downregulated KLF4, which acts as an oncogene in breast cancer. Additionally, the combination was effective in downregulating HDAC activity, especially HDAC2 and HDAC3, which act in concert with KLF4. KLF4 is known to activate hTERT, which is highly activated in breast cancer. Our studies have shown that hTERT is downregulated with the combination. Thus, this combination is very effective in inhibiting breast tumorigenesis without having any toxicity on the normal cells. Future studies will involve assessing KLF4 protein expression in mice tumors as well as the expression of HDAC2, HDAC3 and hTERT in the tumor tissues. Additionally, we will perform experiments to study in depth the *p21* gene, which acts as a switch in KLF4 regulation by studying the *p21* promoter region. We will also study the *hTERT* promoter region to resolve the interaction among KLF4, hTERT, HDAC2 and HDAC3.

## Figures and Tables

**Table 1:** CompuSyn data of MTT values indicate synergism in breast cancer cells.

Cell Lines	SFN	GEN	Average CI
MCF-7	5.0 µM	10.0 µM	0.40468
MCF-7	5.0 µM	15.0 µM	0.29520
MDA-MB-231	5.0 µM	10.0 µM	0.45254
MDA-MB-231	5.0 µM	15.0 µM	0.56832

The table shows the Combination index (CI) values of the 3-(4,5-Dimethylthiazol-2-yl)-2,5-Diphenyltetrazolium Bromide (MTT) assay for different combinations of genistein (GEN) and sulforaphane (SFN) on MCF-7 and MDA-MB-231 cells.

## Supplementary Materials

The following are available online at http://www.mdpi.com/1422-0067/19/6/1754/s1.

## References

[1] DeSantis C.E., Ma J., Goding Sauer A., Newman L.A., Jemal A. (2017). Breast cancer statistics, 2017, racial disparity in mortality by state. CA Cancer J. Clin..

[2] Hardy T.M., Tollefsbol T.O. (2011). Epigenetic diet: Impact on the epigenome and cancer. Epigenomics.

[3] Herceg Z. (2007). Epigenetics and cancer: Towards an evaluation of the impact of environmental and dietary factors. Mutagenesis.

[4] Russo G.L., Vastolo V., Ciccarelli M., Albano L., Macchia P.E., Ungaro P. (2017). Dietary polyphenols and chromatin remodeling. Crit. Rev. Food Sci. Nutr..

[5] Li Y., Meeran S.M., Tollefsbol T.O. (2017). Combinatorial bioactive botanicals re-sensitize tamoxifen treatment in ER-negative breast cancer via epigenetic reactivation of ERα expression. Sci. Rep..

[6] Murrell A. What lies beneath the epigenetic signatures associated with breast cancer and how do we find out?. Proceedings of the UK Breast Cancer Research Symposium.

[7] Park J.-H., Kim S.-H., Lee M.S., Kim M.-S. (2017). Epigenetic modification by dietary factors: Implications in metabolic syndrome. Mol. Aspects Med..

[8] Tahir A.A., Sani N.F.A., Murad N.A., Makpol S., Ngah W.Z.W., Yusof Y.A.M. (2015). Combined ginger extract & Gelam honey modulate Ras/ERK and PI3K/AKT pathway genes in colon cancer HT29 cells. Nutr. J..

[9] Kotecha R., Takami A., Espinoza J.L. (2016). Dietary phytochemicals and cancer chemoprevention: A review of the clinical evidence. Oncotarget.

[10] Chou T.-C. (2010). Drug combination studies and their synergy quantification using the chou-talalay method. Cancer Res..

[11] Xie Q., Bai Q., Zou L.Y., Zhang Q.Y., Zhou Y., Chang H., Yi L., Zhu J.D., Mi M.T. (2014). Genistein inhibits DNA methylation and increases expression of tumor suppressor genes in human breast cancer cells. Genes Chromosomes Cancer.

[12] Tortorella S.M., Royce S.G., Licciardi P.V., Karagiannis T.C. (2015). Dietary sulforaphane in cancer chemoprevention: The role of epigenetic regulation and HDAC inhibition. Antioxid. Redox Signal..

[13] Meeran S.M., Ahmed A., Tollefsbol T.O. (2010). Epigenetic targets of bioactive dietary components for cancer prevention and therapy. Clin. Epigenet..

[14] Li Y., Yuan Y.-Y., Meeran S.M., Tollefsbol T.O. (2010). Synergistic epigenetic reactivation of estrogen receptor-α (ERα) by combined green tea polyphenol and histone deacetylase inhibitor in ERα-negative breast cancer cells. Mol. Cancer.

[15] Royston K.J., Udayakumar N., Lewis K., Tollefsbol T.O. (2017). A novel combination of withaferin A and sulforaphane inhibits epigenetic machinery, cellular viability and induces apoptosis of breast cancer cells. Int. J. Mol. Sci..

[16] Dashwood R.H., Ho E. (2008). Dietary agents as histone deacetylase inhibitors: Sulforaphane and structurally related isothiocyanates. Nutr. Rev..

[17] Traka M.H., Chambers K.F., Lund E.K., Goodlad R.A., Johnson I.T., Mithen R.F. (2008). Involvement of KLF4 in sulforaphane-and iberin-mediated induction of p21 waf1/cip1. Nutr. Cancer.

[18] Rowland B.D., Peeper D.S. (2006). KLF4, p21 and context-dependent opposing forces in cancer. Nat. Rev. Cancer.

[19] Wei D., Kanai M., Huang S., Xie K. (2005). Emerging role of KLF4 in human gastrointestinal cancer. Carcinogenesis.

[20] Yang Y., Goldstein B.G., Chao H.-H., Katz J. (2005). KLF4 and KLF5 regulate proliferation, apoptosis and invasion in esophageal cancer cells. Cancer Biol. Ther..

[21] Yu F., Li J., Chen H., Fu J., Ray S., Huang S., Zheng H., Ai W. (2011). Kruppel-like factor 4 (KLF4) is required for maintenance of breast cancer stem cells and for cell migration and invasion. Oncogene.

[22] Okuda H., Xing F., Pandey P.R., Sharma S., Watabe M., Pai S.K., Mo Y.-Y., Iiizumi-Gairani M., Hirota S., Liu Y. (2013). Mir-7 suppresses brain metastasis of breast cancer stem-like cells by modulating KLF4. Cancer Res..

[23] Pandya A.Y., Talley L.I., Frost A.R., Fitzgerald T.J., Trivedi V., Chakravarthy M., Chhieng D.C., Grizzle W.E., Engler J.A., Krontiras H. (2004). Nuclear localization of KLF4 is associated with an aggressive phenotype in early-stage breast cancer. Clin. Cancer. Res..

[24] Li R., Liang J., Ni S., Zhou T., Qing X., Li H., He W., Chen J., Li F., Zhuang Q. (2010). A mesenchymal-to-epithelial transition initiates and is required for the nuclear reprogramming of mouse fibroblasts. Cell Stem Cell.

[25] Ray A., Alalem M., Ray B.K. (2013). Loss of epigenetic kruppel-like factor 4 histone deacetylase (KLF-4-HDAC)-mediated transcriptional suppression is crucial in increasing vascular endothelial growth factor (VEGF) expression in breast cancer. J. Biol. Chem..

[26] Hsieh M.-H., Chen Y.-T., Chen Y.-T., Lee Y.-H., Lu J., Chien C.-L., Chen H.-F., Ho H.-N., Yu C.-J., Wang Z.-Q. (2017). Parp1 controls KLF4-mediated telomerase expression in stem cells and cancer cells. Nucleic Acids Res..

[27] Kim S.-H., Wan Y., Singh S.V. Breast cancer stem cell inhibition by benzyl isothiocyanate is hampered by induction of kruppel-like factor 4. Proceedings of the AACR Annual Meeting.

[28] Chou T., Martin N. (2005). A computer program for quantitation of synergism and antagonism in drug combinations, and the determination of IC50 and ED50 and LD50 values. CompuSyn for Drug Combinations: PC Software and User’s Guide.

[29] Fu J., Chou T.-C. Abstract 4554A: Simple, efficient, and quantitative approach for determination of synergism, additive effect, and antagonism of drugs in vivo using combination index method: A proposition for clinical protocol design and regulatory synergy claims. Proceedings of the AACR Annual Meeting.

[30] Chou T.-C. (2008). Preclinical versus clinical drug combination studies. Leuk. Lymphoma.

[31] Hu W., Jia Y., Xiao X., Lv K., Chen Y., Wang L., Luo X., Liu T., Li W., Li Y. (2016). KLF4 downregulates hTERT expression and telomerase activity to inhibit lung carcinoma growth. Oncotarget.

[32] Key T.J., Schatzkin A., Willett W.C., Allen N.E., Spencer E.A., Travis R.C. (2004). Diet, nutrition and the prevention of cancer. Public Health Nutr..

[33] Esteller M., Herman J.G. (2002). Cancer as an epigenetic disease: DNA methylation and chromatin alterations in human tumours. J. Pathol..

[34] Paul B., Barnes S., Demark-Wahnefried W., Morrow C., Salvador C., Skibola C., Tollefsbol T.O. (2015). Influences of diet and the gut microbiome on epigenetic modulation in cancer and other diseases. Clin. Epigenet..

[35] Youlden D.R., Cramb S.M., Yip C.H., Baade P.D. (2014). Incidence and mortality of female breast cancer in the Asia-pacific region. Cancer Biol. Med..

[36] Ferrini K., Ghelfi F., Mannucci R., Titta L. (2015). Lifestyle, nutrition and breast cancer: Facts and presumptions for consideration. Ecancermedicalscience.

[37] Grosso G., Bella F., Godos J., Sciacca S., Del Rio D., Ray S., Galvano F., Giovannucci E.L. (2017). Possible role of diet in cancer: Systematic review and multiple meta-analyses of dietary patterns, lifestyle factors, and cancer risk. Nutr. Rev..

[38] Meeran S.M., Patel S.N., Li Y., Shukla S., Tollefsbol T.O. (2012). Bioactive dietary supplements reactivate ER expression in ER-negative breast cancer cells by active chromatin modifications. PLoS ONE.

[39] Sinha S., Shukla S., Khan S., Tollefsbol T.O., Meeran S.M. (2015). Epigenetic reactivation of p21CIP1/WAF1 and KLOTHO by a combination of bioactive dietary supplements is partially ERα-dependent in ERα-negative human breast cancer cells. Mol. Cell. Endocrinol..

[40] Kikuno N., Shiina H., Urakami S., Kawamoto K., Hirata H., Tanaka Y., Majid S., Igawa M., Dahiya R. (2008). Genistein mediated histone acetylation and demethylation activates tumor suppressor genes in prostate cancer cells. Int. J. Cancer.

[41] Majid S., Dar A.A., Ahmad A.E., Hirata H., Kawakami K., Shahryari V., Saini S., Tanaka Y., Dahiya A.V., Khatri G. (2009). BTG3 tumor suppressor gene promoter demethylation, histone modification and cell cycle arrest by genistein in renal cancer. Carcinogenesis.

[42] Paul B., Royston K.J., Li Y., Stoll M.L., Skibola C.F., Wilson L.S., Barnes S., Morrow C.D., Tollefsbol T.O. (2017). Impact of genistein on the gut microbiome of humanized mice and its role in breast tumor inhibition. PLoS ONE.

[43] Ganai S.A. (2016). Histone deacetylase inhibitor sulforaphane: The phytochemical with vibrant activity against prostate cancer. Biomed. Pharmacother..

[44] Tarapore R.S., Siddiqui I.A., Mukhtar H. (2011). Modulation of wnt/β-catenin signaling pathway by bioactive food components. Carcinogenesis.

[45] Li Y., Zhang T., Korkaya H., Liu S., Lee H.-F., Newman B., Yu Y., Clouthier S.G., Schwartz S.J., Wicha M.S. (2010). Sulforaphane, a dietary component of broccoli/broccoli sprouts, inhibits breast cancer stem cells. Clin. Cancer. Res..

[46] Meeran S.M., Patel S.N., Tollefsbol T.O. (2010). Sulforaphane causes epigenetic repression of hTERT expression in human breast cancer cell lines. PLoS ONE.

[47] West A.C., Johnstone R.W. (2014). New and emerging HDAC inhibitors for cancer treatment. J. Clin. Investig..

[48] Myzak M.C., Karplus P.A., Chung F.-L., Dashwood R.H. (2004). A novel mechanism of chemoprotection by sulforaphane: Inhibition of histone deacetylase. Cancer Res..

[49] Sasamura H., Takahashi A., Yuan J., Kitamura H., Masumori N., Miyao N., Itoh N., Tsukamoto T. (2004). Antiproliferative and antiangiogenic activities of genistein in human renal cell carcinoma. Urology.

[50] Majid S., Kikuno N., Nelles J., Noonan E., Tanaka Y., Kawamoto K., Hirata H., Li L.C., Zhao H., Okino S.T. (2008). Genistein induces the p21WAF1/CIP1 and p16INK4a tumor suppressor genes in prostate cancer cells by epigenetic mechanisms involving active chromatin modification. Cancer Res..

[51] Fahey J.W., Zhang Y., Talalay P. (1997). Broccoli sprouts: An exceptionally rich source of inducers of enzymes that protect against chemical carcinogens. Proc. Natl. Acad. Sci. USA.

[52] Atwell L.L., Hsu A., Wong C.P., Stevens J.F., Bella D., Yu T.-W., Pereira C.B., Löhr C.V., Christensen J.M., Dashwood R.H. (2015). Absorption and chemopreventive targets of sulforaphane in humans following consumption of broccoli sprouts or a myrosinase-treated broccoli sprout extract. Mol. Nutr. Food Res..

[53] Hu R., Khor T.O., Shen G., Jeong W.-S., Hebbar V., Chen C., Xu C., Reddy B., Chada K., Kong A.-N.T. (2006). Cancer chemoprevention of intestinal polyposis in ApcMin/+ mice by sulforaphane, a natural product derived from cruciferous vegetable. Carcinogenesis.

[54] Lamartiniere C.A., Cotroneo M.S., Fritz W.A., Wang J., Mentor-Marcel R., Elgavish A. (2002). Genistein chemoprevention: Timing and mechanisms of action in murine mammary and prostate. J. Nutr..

[55] Jin Z., MacDonald R.S. (2002). Soy isoflavones increase latency of spontaneous mammary tumors in mice. J. Nutr..

[56] Ferralli J., Chiquet-Ehrismann R., Degen M. (2016). KLF4A stimulates breast cancer cell proliferation by acting as a KLF4 antagonist. Oncotarget.

[57] Ema M., Mori D., Niwa H., Hasegawa Y., Yamanaka Y., Hitoshi S., Mimura J., Kawabe Y.-I., Hosoya T., Morita M. (2008). Krüppel-like factor 5 is essential for blastocyst development and the normal self-renewal of mouse ESCs. Cell Stem Cell.

[58] Wong C.W., Hou P.S., Tseng S.F., Chien C.L., Wu K.J., Chen H.F., Ho H.N., Kyo S., Teng S.C. (2010). Krüppel-like transcription factor 4 contributes to maintenance of telomerase activity in stem cells. Stem Cells.

[59] Pons D.G., Nadal-Serrano M., Blanquer-Rossello M., Sastre-Serra J., Oliver J., Roca P. (2014). Genistein modulates proliferation and mitochondrial functionality in breast cancer cells depending on ERalpha/ERbeta ratio. J. Cell. Biochem..

[60] Yori J.L., Seachrist D.D., Johnson E., Lozada K.L., Abdul-Karim F.W., Chodosh L.A., Schiemann W.P., Keri R.A. (2011). Krüppel-like factor 4 inhibits tumorigenic progression and metastasis in a mouse model of breast cancer. Neoplasia.

[61] Su Y., Simmen R.C. (2008). Soy isoflavone genistein upregulates epithelial adhesion molecule E-cadherin expression and attenuates β-catenin signaling in mammary epithelial cells. Carcinogenesis.

[62] Singh S.V., Warin R., Xiao D., Powolny A.A., Stan S.D., Arlotti J.A., Zeng Y., Hahm E.-R., Marynowski S.W., Bommareddy A. (2009). Sulforaphane inhibits prostate carcinogenesis and pulmonary metastasis in tramp mice in association with increased cytotoxicity of natural killer cells. Cancer Res..

[63] Shen G., Khor T.O., Hu R., Yu S., Nair S., Ho C.-T., Reddy B.S., Huang M.-T., Newmark H.L., Kong A.-N.T. (2007). Chemoprevention of familial adenomatous polyposis by natural dietary compounds sulforaphane and dibenzoylmethane alone and in combination in ApcMin/+ mouse. Cancer Res..

[64] Fritz W.A., Wang J., Eltoum I.-E., Lamartiniere C.A. (2002). Dietary genistein downregulates androgen and estrogen receptor expression in the rat prostate. Mol. Cell. Endocrinol..

[65] Lee A.V., Oesterreich S., Davidson N.E. (2015). Mcf-7 cells—Changing the course of breast cancer research and care for 45 years. JNCI J. Natl. Cancer Inst..

[66] Lacroix M., Leclercq G. (2004). Relevance of breast cancer cell lines as models for breast tumours: An update. Breast Cancer Res. Treat..

[67] Neve R.M., Chin K., Fridlyand J., Yeh J., Baehner F.L., Fevr T., Clark L., Bayani N., Coppe J.-P., Tong F. (2006). A collection of breast cancer cell lines for the study of functionally distinct cancer subtypes. Cancer Cell.

[68] Chen H., Landen C.N., Li Y., Alvarez R.D., Tollefsbol T.O. (2013). Enhancement of cisplatin-mediated apoptosis in ovarian cancer cells through potentiating G2/M arrest and p21 upregulation by combinatorial epigallocatechin gallate and sulforaphane. J. Oncol..

[69] Li Y., Meeran S.M., Patel S.N., Chen H., Hardy T.M., Tollefsbol T.O. (2013). Epigenetic reactivation of estrogen receptor-α (ERα) by genistein enhances hormonal therapy sensitivity in ERα-negative breast cancer. Mol. Cancer.

