# Supplementary material for: The Effects of Combinatorial Genistein and Sulforaphane in Breast Tumor Inhibition: Role in Epigenetic Regulation
Source: Int J Mol Sci. 2018 Jun 13;19(6):1754. doi: 10.3390/ijms19061754 (PMC6032337; doi:10.3390/ijms19061754)
Supplement: Supplementary file 1 [file ijms-19-01754-s001.pdf]

Article

The Effects of Combinatorial Genistein and Sulforaphane in Breast Tumor Inhibition: Role in Epigenetic Regulation

Bidisha Paul [1], Yuanyuan Li [2,3,4] and Trygve O. Tollefsbol [1,2,3,5,6,*]

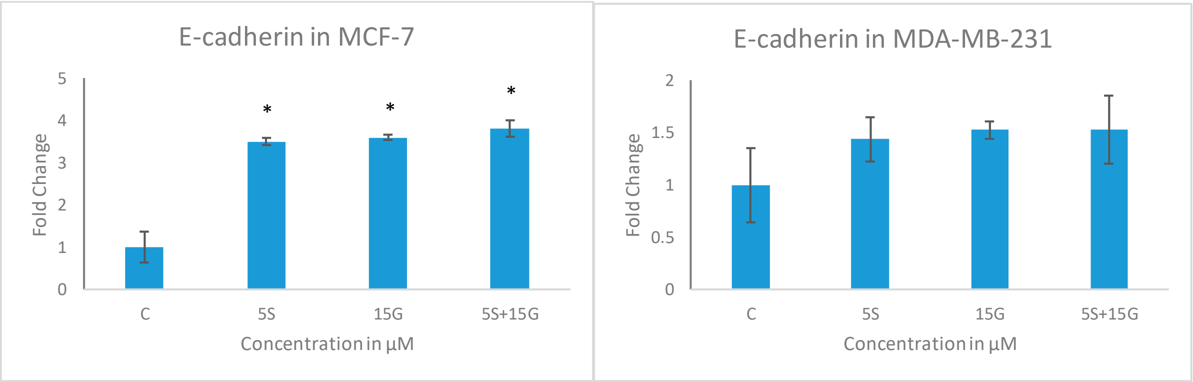

Figure S1. E-cadherin RT-PCR.
